# Transcriptome Responses to Different Salinity Conditions in *Litoditis marina,* Revealed by Long-Read Sequencing

**DOI:** 10.3390/genes15030317

**Published:** 2024-02-28

**Authors:** Pengchi Zhang, Beining Xue, Hanwen Yang, Liusuo Zhang

**Affiliations:** 1 CAS and Shandong Province Key Laboratory of Experimental Marine Biology, Institute of Oceanology, Chinese Academy of Sciences, Qingdao 266071, China; zhangpengchi@qdio.ac.cn (P.Z.); xuebeining18@mails.ucas.edu.cn (B.X.); hanwen.yang.1@slu.edu (H.Y.); 2 Laboratory of Marine Biology and Biotechnology, Qingdao National Laboratory for Marine Science and Technology, Qingdao 266237, China; 3 Center for Ocean Mega-Science, Chinese Academy of Sciences, 7 Nanhai Road, Qingdao 266071, China; 4 University of Chinese Academy of Sciences, Beijing 100049, China

**Keywords:** marine nematode, *Litoditis marina*, ONT long-read sequencing technology, salinity, alternative splicing, alternative polyadenylation (APA)

## Abstract

The marine nematode *Litoditis marina* is widely distributed in intertidal zones around the globe, yet the mechanisms underlying its broad adaptation to salinity remain elusive. In this study, we applied ONT long-read sequencing technology to unravel the transcriptome responses to different salinity conditions in *L. marina*. Through ONT sequencing under 3‰, 30‰ and 60‰ salinity environments, we obtained 131.78 G clean data and 26,647 non-redundant long-read transcripts, including 6464 novel transcripts. The DEGs obtained from the current ONT lrRNA-seq were highly correlated with those identified in our previously reported Illumina short-read RNA sequencing data. When we compared the 30‰ to the 3‰ salinity condition, we found that GO terms such as oxidoreductase activity, cation transmembrane transport and ion transmembrane transport were shared between the ONT lrRNA-seq and Illumina data. Similarly, GO terms including extracellular space, structural constituents of cuticle, substrate-specific channel activity, ion transport and substrate-specific transmembrane transporter activity were shared between the ONT and Illumina data under 60‰ compared to 30‰ salinity. In addition, we found that 79 genes significantly increased, while 119 genes significantly decreased, as the salinity increased. Furthermore, through the GO enrichment analysis of 214 genes containing DAS, in 30‰ compared to 3‰ salinity, we found that GO terms such as cellular component assembly and coenzyme biosynthetic process were enriched. Additionally, we observed that GO terms such as cellular component assembly and coenzyme biosynthetic process were also enriched in 60‰ compared to 30‰ salinity. Moreover, we found that 86, 125, and 81 genes that contained DAS were also DEGs, in comparisons between 30‰ and 3‰, 60‰ and 30‰, and 60‰ and 3‰ salinity, respectively. In addition, we demonstrated the landscape of alternative polyadenylation in marine nematode under different salinity conditions This report provides several novel insights for the further study of the mechanisms by which euryhalinity formed and evolved, and it might also contribute to the investigation of salinity dynamics induced by global climate change.

## 1. Introduction

Sufficient sensation, appropriate responses, and an adaptation to different salinity conditions are essential for all living organisms’ development, reproduction, and survival. A euryhaline nematode, *L. marina*, inhabiting, globally, the littoral zone of coasts and estuaries, is an emerging model animal for osmoregulation studies. Based on Illumina RNA sequencing, our recent report showed that its expression of transthyretin-like (TTL) genes was significantly upregulated, while many of its neurotransmitter receptor and ion transporter genes were repressed in both hyposaline and hypersaline environments [1]. It has been described that fatty acid biosynthesis genes, tubulins and intraflagellar transport genes were increased in *L. marina* under hyposaline conditions, in contrast to the finding that its expression of collagen genes was significantly accelerated in hypersaline environments [1]. However, the mechanisms underlying how invertebrate animals perceive various osmotic environments, transduce signals and adapt to dynamic salinity conditions remain poorly understood.

Oxford Nanopore Technologies (ONT) long-read sequencing (lrRNA-seq) has emerged as a powerful tool in the transcriptomics field, providing several advantages over Illumina short-read sequencing, such as alternative splicing (AS) identification, full-length transcript detection, untranslated regions (UTRs) (5′ UTR and 3′ UTR) and alternative polyadenylation (APA) analysis [2,3,4,5,6,7,8]. It was reported that ONT lrRNA-seq was applied to the gills and eyes of the migratory fish *Coilia nasus* under both hypo- and hypersaline conditions, demonstrating its differentially expressed genes (DEGs) and differentially expressed transcripts (DETs), respectively [9,10].

Alternative splicing (AS) can identify the skipping exon (SE), mutually exclusive exon (MX), alternative 5′ splice site (A5), alternative 3′ splice site (A3), retained intron (RI), alternative first exon (AF), and alternative last exon (AL), which greatly enriches the diversity of mRNAs and proteins [11,12,13,14,15,16,17,18]. It has been reported that an isoform with intron 1 was retained in Cox-1 regulated human intestinal epithelial osmoregulation in a cyclooxygenase-mediated manner [19]. It has been described that the longer isoform of *SLC2a5* was induced under both low- and high-salinity stresses, and that different isoforms of *Cyb5r3* dominated in different salinity environments in *Ciona savignyi* [20]. Enhanced 3′ UTR shortening through alternative polyadenylation (APA) was induced in *C. savignyi* under both hypo- and hypersaline conditions [20]. APA usually occurs in 3′ UTRs and influences the stability of mRNAs or/and proteins [21].

In this study, ONT lrRNA-seq was deployed to reveal the transcriptome responses to different salinity conditions of *L. marina* for the first time. We demonstrated its DEGs, DETs, and DAS events, as well as the landscape of its 3′ UTR and APA between 3‰, 30‰ and 60‰ salinity conditions.

## 2. Material and Methods

### 2.1. Worms

Marine nematode *L. marina*: HQ1, the wild-type strain isolated from Huiquan Bay, Qingdao, was used in this study. Its cultivation follows our previous publications [1,22,23].

### 2.2. Sample Collection and ONT lrRNA Sequencing Library Preparation

HQ1 worms were cultured in 9 cm seawater NGM (SW-NGM), seeded with 15X OP50, at 20 °C. When a large number of eggs were laid on the culture plates, adult worms were collected by rinsing the plates with sterilized seawater. Subsequently, the eggs on the plates were rinsed into a collection tube and bleached with Worm Bleaching Solution [1] to obtain sterile eggs, which were incubated in sterilized seawater overnight to hatch as synchronized L1 larvae. These synchronized L1 larvae were transferred to plates seeded with 1000 μL OP50 for 3 h, with three replicates for each treatment (3‰, 30‰, and 60‰ salinity conditions, respectively). After 3 h of feeding, excess bacteria were rinsed off with M9, and the worms were immediately collected. Subsequently, the collected worms were frozen in liquid nitrogen and stored at −80 °C [1]. The total RNA of each sample was extracted using Trizol (Invitrogen). For each treatment, three biological replicates were prepared, resulting in a total of nine RNA libraries. The libraries were prepared using the Ligation Sequencing Kit 1D (PM) (SQK-LSK109, Nanopore, Oxford, UK), following the manufacturer’s recommendations. Subsequently, the RNA libraries were sequenced on a PromethION48 (Nanopore) using the PromethION Flow Cell (R9 Version) (FLO-PRO002, Nanopore).

### 2.3. Transcriptome Construction from ONT Reads

IsoQuant 3.3.1 was deployed for transcriptome construction using all ONT reads available, with *L. marina* Genome v1.0 and Geneset v1.0 as references (-d nanopore -complete_genedb --count_exons --transcript_quantification with_inconsistent --no_secondary) [24]. The GTF files with the discovered expressed transcript (transcript_models.gtf) from each salinity condition were then compared with Geneset v1.0 using GffCompare v0.12.6 [25]. All transcripts were then classified into various structural categories using SQANTI3 v5.1.2 and manually curated for further analysis [26]. Only transcripts that were not categorized as ‘fusion’ or ‘full splice match’ or ‘incomplete splice match’, and not labeled as ‘bite’ or an RT switching artifact were considered to be putative novel transcripts. These putative novel transcripts, combined with known transcripts from Geneset v1.0, were then used for quantification using minimap2 v2.17-r941 and the ‘mapping-based mode’ of Salmon v0.15.0 (minimap2: -ax splice. Salmon: -noErrorModel, -l U.) [27,28]. Transcripts with more than 5 reads across all samples of each condition (0.3%, 3% or 6%) were retained as novel transcripts.

### 2.4. Gene and Transcript Abundance Estimation

ONT reads were aligned to all known and novel transcripts using minimap2 again, with the same parameters. Alignments were filtered using samtools v1.7 to remove unmapped reads and secondary alignments (-F 260) [29]. Gene and transcript abundance were estimated using Salmon v0.15.0 with the same parameters but using all known and novel transcripts as reference.

### 2.5. Differential Expression Analysis

The quantified raw read counts of the genes and transcripts were obtained from R package TxImport v1.26.1 [30]. For low-expression filtering and normalization, we performed filterByExpr and the weighted trimmed mean of M-values (TMM) method from the R package edgeR v3.40.2, using default arguments on the raw read counts [31]. Then, differentially expressed genes (DEGs) and transcripts (DETs) analyses were performed using the quasi-likelihood pipeline of edgeR. DEGs or DETs with an adjusted *p*-value (padj) < 0.05 and a log_2_ fold change > 0.558 were identified as significantly differentially expressed genes or transcripts, respectively. The R version used in this study is 4.2.2.

### 2.6. Alternative Splicing Analysis

Alternative splicing analysis was performed with SUPPA2 (V2.3), based on the transcript quantification results of Salmon [18]. All potential AS events were generated from all known and novel transcripts (generateEvents, -e SE SS MX RI FL -f ioe). Then, ‘diffSplice’ was used for identifying differential alternative splicing events using the ‘psiPerEvent’ for psi values (diffSplice, --method empirical). AS events with |ΔPSI| > 0.1 and a *p*-value < 0.05 were considered statistically significant.

### 2.7. Alternative Polyadenylation Analysis

All ONT reads were mapped to *L. marina* Genome v1.0 using minimap2 (-ax splice). Alignments were then filtered and sorted using samtools and Sambamba v0.6.7-pre1 (samtools, -F260; Sambamba, sort) [32]. BAM files were then converted to BedGraph files, using BEDTools, for further analysis [33]. APAtrap was used for identifying putative APA sites and differentially expressed APA genes [34]. First, a gene model file, in 12-column bed format (bed12), was constructed from the GTF file containing the longest isoform (of all known and novel transcripts) using AGAT v0.4.0. Putative novel 3′ UTRs or 3′ UTR extensions were identified using ‘identifyDistal3UTR’ on all alignments with default parameters. Next, ‘predictAPA’ and R package deAPA v1.0 were run with default parameters for each comparison group. Only genes with an absolute r >0.1, PD > 0.2 and adjusted *p*-value < 0.05 were considered to be significantly different in APA site usage.

### 2.8. Analysis of 3′ UTR

Information on the 3′ UTR of each transcript was extracted from the output of the ‘IsoAnnotLite’ of Sqanti3. A Poly(A) motif list for Poly(A) motif detection (polyA_motif) and the distance (polyA_dist) to the closest polyA motif calculation were obtained from PolyASite (*Caenorhabditis elegans*: v2.0 (WBcel235) [35]. Sequence logos of the 3′ UTRs were produced using the R package ggseqlogo v0.1, based on the output of ‘IsoAnnotLite’.

### 2.9. Gene Set and Pathway Enrichment Analyses

The R package clusterProfiler v4.6.2 was used to perform KEGG and GO enrichment analyses on given sets of genes. The KEGG and GO functional annotations were retrieved from *L. marina* Geneset v1.0 [1,22,23].

## 3. Result

### 3.1. Overview of ONT lrRNA Sequencing in Different Salinity Conditions

Approximately 39.41 G, 45.25 G, and 47.12 G of ONT data were obtained, comprising 5.34, 5.01, and 6.25 million reads, under the 3‰, 30‰, and 60‰ salinity conditions, respectively (Table 1). 13,769, 14,098 and 13,920 isoforms were detected in the 3‰, 30‰, and 60‰ salinity conditions, respectively, of which 3161, 3538, and 3226 were novel isoforms identified in this study (Table 1).

### 3.2. Differentially Expressed Genes (DEGs) Analysis

**Figure 1:**
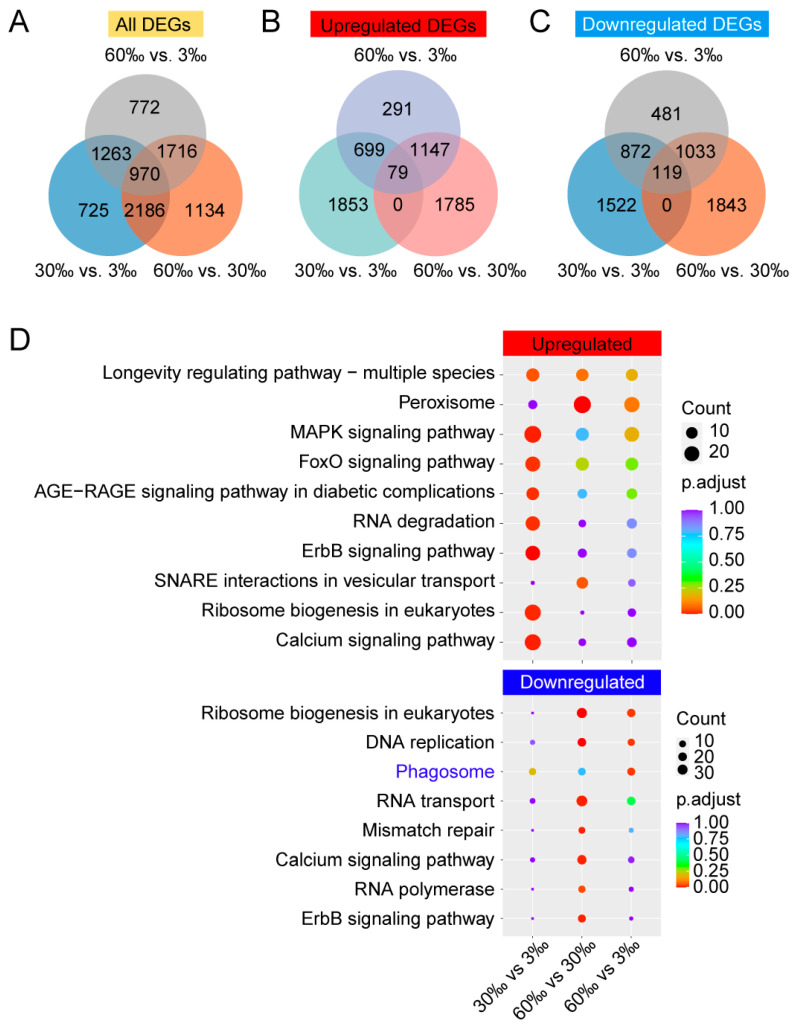
The characterization of differentially expressed genes (DEGs) from *L. marina* lrRNA–Seq. (**A**) Venn diagrams illustrating common DEGs between different salinity conditions. (**B**) Venn diagrams illustrating upregulated DEGs across different salinity conditions. (**C**) Venn diagrams illustrating downregulated DEGs across different salinity conditions. (**D**) KEGG enrichment analysis between different salinity conditions.

We found 5144, 6006, and 4721 DEGs, when comparisons were made of the 30‰ vs. 3‰, 60‰ vs. 30‰, and 60‰ vs. 3‰ salinity conditions, respectively (Figure 1A). Of the above-mentioned DEGs, there were 2631, 3011, and 2216 upregulated DEGs, of which 79 were shared by all three conditions (Figure 1B), and 2513, 2995, and 2505 downregulated DEGs with 119 overlapping genes across the three salinity conditions (Figure 1C). Details of the significant differentially expressed genes (DEGs) are presented in Supplementary Table S1. Through the KEGG analysis of the upregulated DEGs, we found that pathways such as the ErbB signaling pathway, MAPK signaling pathway, and calcium signaling pathway were significantly enriched in the 30‰ compared to the 3‰ condition (Figure 1D, Supplementary Table S2). In contrast, pathways such as peroxisome and SNARE interactions in vesicular transport were significantly enriched in the 60‰ compared to the 30‰ condition (Figure 1D, Supplementary Table S3). When KEGG analysis was applied to the downregulated DEGs, we observed that pathways including the ribosome biogenesis in eukaryotes, DNA replication, and calcium signaling pathways were significantly enriched in the 60‰ compared to the 30‰ condition (Figure 1D, Supplementary Table S4).

The GO analysis revealed that the expression of genes belonging to terms such as intracellular signal transduction, hydrolase activity—acting on acid anhydrides, and motor activity was significantly upregulated under the 30‰ compared to the 3‰ condition (Supplementary Figure S2, Supplementary Table S5). In contrast, the expression of genes associated with terms such as extracellular space, iron ion binding, and oxidoreductase activity—acting on single donors with the incorporation of molecular oxygen—were significantly downregulated (Supplementary Figure S2, Supplementary Table S6) under the 30‰ compared to the 3‰ condition. Among the upregulated DEGs, we found that GO terms including extracellular space, the mitochondrial inner membrane, and the structural constituent of cuticle were significantly enriched in the 60‰ compared to the 30‰ condition (Supplementary Figure S3 and Supplementary Table S7). In contrast, among the downregulated DEGs, we found that GO terms such as ion transport, ribosome biogenesis and DNA packaging were significantly enriched in the 60‰ compared to the 30‰ condition (Supplementary Figure S3 and Supplementary Table S8).

For the 79 upregulated DEGs shared by all the three comparisons between salinity conditions (Figure 1B), we performed a GO analysis and found that GO terms such as pyridoxal phosphate binding, the structural constituent of cuticle, and carbohydrate binding were enriched (Supplementary Table S9). For the 119 downregulated DEGs that overlapped across all three salinity conditions (Figure 1C), we observed that GO terms including acid phosphatase activity, hydrolase activity—acting on ester bonds, and ion transport were enriched (Supplementary Table S10).

### 3.3. Analysis of Differential Alternative Splicing (DAS) and Differentially Expressed Genes (DEGs)

**Figure 2:**
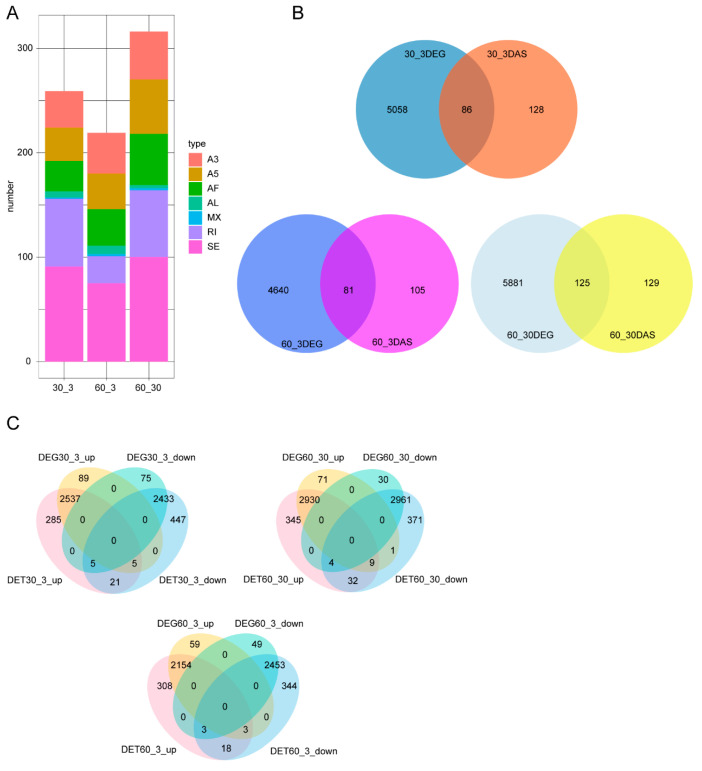
The alternative splicing (AS) of different salinity conditions. (**A**) Composition of DAS events in different salinity conditions. (**B**) Venn diagram depicting the overlap of DEGs and DAS events of different salinity conditions. (**C**) Venn diagram depicting the overlap of DEGs and DETs of different salinity conditions.

We observed 259, 316, and 219 DASs, belonging to 214, 254, and 186 genes, when comparisons were made of the 30‰ vs. 3‰, 60‰ vs. 30‰, and 60‰ vs. 3‰ salinity conditions, respectively (Figure 2A, Supplementary Table S11). The GO enrichment analysis of the 214 genes containing DASs between 30‰ and 3‰ salinity revealed an enrichment in terms such as cellular component assembly, coenzyme biosynthetic process, and protein homo-oligomerization (Supplementary Table S12). For the 60‰ vs. 30‰ comparison, an enrichment was observed in GO terms including cellular component assembly, coenzyme biosynthetic process, and vacuolar transport (Supplementary Table S13). In addition, between the 60‰ and 3‰ salinity conditions, we found that GO terms such as cellular macromolecular complex assembly, intracellular organelle part, and the mediator complex were enriched (Supplementary Table S14).

Next, we asked whether there was overlapping between the DASs and DEGs; we found that 86, 125, and 81 genes that underwent DAS events belonged to DEGs, among our comparisons of 30‰ vs. 3‰, 60‰ vs. 30‰, and 60‰ vs. 3‰ salinity, respectively (Figure 2B). The overlapping 86 DAS genes between the 30‰ and 3‰ conditions are primarily enriched in GO terms such as the nucleus, ion transport, and aromatic compound biosynthetic process (Supplementary Table S15). Similarly, the overlapping 125 DAS genes between 60‰ and 30‰ salinity are mainly enriched in GO terms such as the aromatic compound biosynthetic process, signal transduction, and the ion transmembrane transporter (Supplementary Table S16). In addition, the overlapping 81 DAS genes between the 60‰ and 3‰ groups are primarily enriched in GO terms such as intracellular membrane-bounded organelles, nitrogen compound transport, and amide transport (Supplementary Table S17). By comparing DEGs and DETs, we observed that the majority of DEGs exhibited a differential expression at the transcript level, while some transcripts showed significant expression differences without causing significant differences in gene expression (Figure 2C).

### 3.4. Landscape of 3′ UTRs

**Figure 3:**
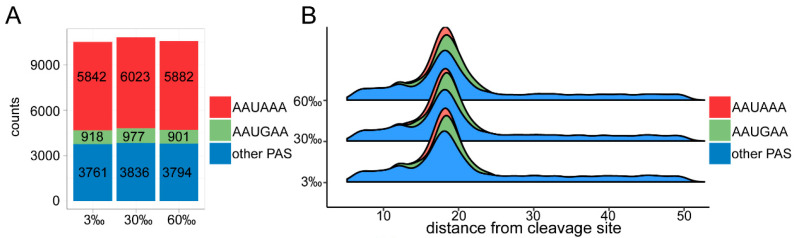

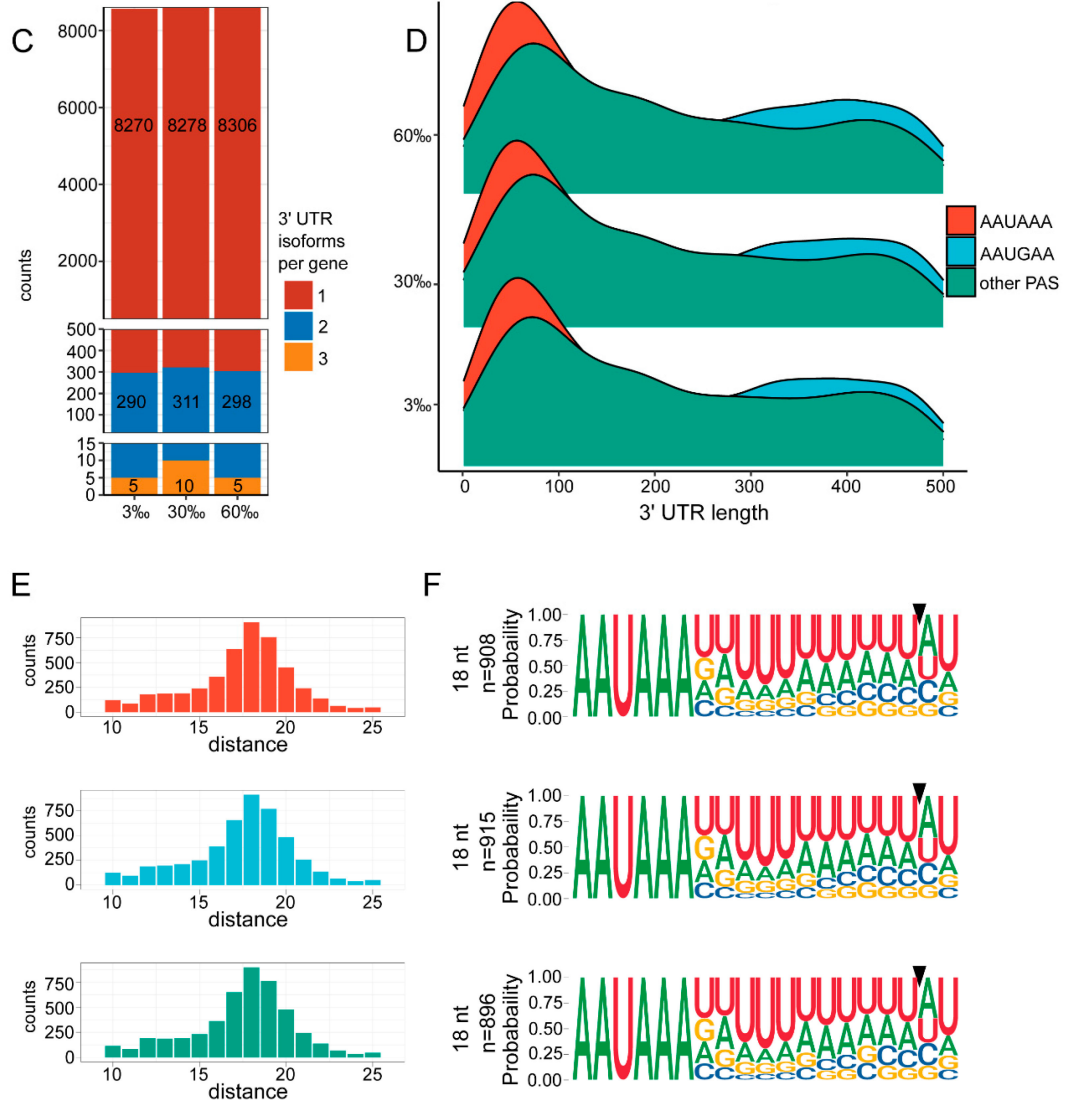
3′ UTR landscape under different salinity conditions. (**A**) Number of PAS elements in 3′ UTRs of *L. marina* under 3‰ salinity, 30‰ salinity and 60‰ salinity. The number of PAS elements in 3′ UTRs without a PAS is not shown. (**B**) Distance from cleavage site to PAS motif in 3′ UTRs of *L. marina* under 3‰ salinity, 30‰ salinity, and 60‰ salinity. The distance is defined as the length between the first nucleotide in the PAS motif and the last nucleotide of the cleavage site. (**C**) Number of isoforms (transcripts) with different PAS motifs in the 3′ UTRs of *L. marina* under salinity stress. 3′ UTRs without a PAS are not shown. (1: only 1 type of PAS motif in 3′ UTR of each gene; 2: 2 types of PAS motif in 3′ UTR of each gene; 3: 3 types of PAS motif in 3′ UTR of each gene.) (**D**) 3′ UTR lengths of *L. marina* under 3‰ salinity, 30‰ salinity, and 60‰ salinity. Lengths of isoforms are shown. (**E**) Distance from cleavage site to PAS motif of genes with only the canonical motif AAUAAA. From top to bottom: 3‰ salinity, 30‰ salinity, and 60‰ salinity. (**F**) Sequence logos representing 18 nt (median distance from cleavage site to PAS motif) downstream of PAS motif in 3′ UTRs under salinity stress. Only the canonical motif AAUAAA is shown. Black triangle indicates the putative cleavage site. From top to bottom: 3‰ salinity, 30‰ salinity, and 60‰ salinity.

To investigate the global 3′ UTR landscape changes in different salinity environments, we analyzed the PAS element, cleavage sites, and the 3′ UTR’s length. As expected, the canonical PAS element “AAUAAA” was the most abundant motif occurring under all three salinity conditions, whereas “AAUGAA” was the most common variant of the PAS element (Figure 3A). Then, we compared the distances of the three types of PAS element (AAUAAA, AAUGAA, and other PAS) to their corresponding cleavage sites under different salinities. For both the “AAUAAA” and “AAUGAA” categories, the median distances were 18 nt under all salinity conditions, while they were 19 nt for isoforms with “other PAS” motifs (Figure 3B). However, in terms of their average distances, isoforms with the above three categories of PAS motifs varied slightly (Supplementary Table S18). Then, we compared the number of isoforms (transcripts) of each gene with different PAS elements in the 3′ UTRs under different salinity stresses based on their SQANTI result (Figure 3C). In total, 59.0% of genes with detected PAS motifs showed no change in their type of motif across salinities, about 39.9% genes showed changes in at least one comparison and only 1.1% genes showed changes in all comparisons (30% vs. 30‰, 60% vs. 30%, and 60% vs. 30‰) (Supplementary Table S18). In addition, the 3′ UTRs under 30‰ salinity were longer than those under both 3‰ and 60% salinity (Figure 3D, Supplementary Table S18). Isoforms with the canonical PAS motif also had a shorter 3′ UTR compared to those with other motifs (Supplementary Table S18). Additionally, varied 3′ UTR lengths were also observed among transcripts with different types of PAS motifs, indicating a possible relationship between PAS usage and 3′ UTR length (Figure 3D, Supplementary Table S18). Next, we analyzed the conservation in the sequence of the buffer region near the cleavage site. Since the canonical PAS motif was the most abundant type of PAS in this study, we focused on genes with only the canonical “AAUAAA” and found that most genes had a median distance from their cleavage site to PAS motif of 18 nt (Figure 3E). Then, we plotted the nucleotides 10 to 40 nt downstream of the canonical PAS element and found profound similarities across salinity conditions (Figure 3F, Supplementary Figures S7–S15). In line with a previous study on *C. elegans*, the terminal UA dinucleotide can also be found at the cut sites in some UTRs, e.g., those of genes with a distance from their canonical PAS motif to their cleavage site of 18 nt [36]. However, dinucleotides including UU were also identified.

### 3.5. APA Analysis

**Figure 4:**
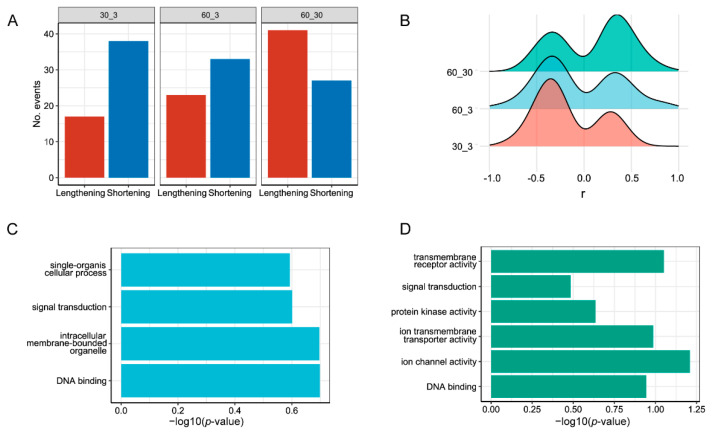
Alternative polyadenylation (APA) under different salinity conditions. (**A**) Numbers of the lengthening and shortening of 3′ UTRs under different salinities. FDR < 0.05, PD index > 0.2, r > 0.1 or <−0.1. 30_3: 30‰ vs. 3‰; 60_3: 60‰ vs. 3‰; 60_30: 60‰ vs. 30‰. (**B**) Distribution of Pearson correlation coefficient (r) under different salinities. FDR < 0.05, PD index > 0.2, r > 0.1 or <−0.1. (**C**) Enriched GO terms from significantly differential APA genes in 30‰ compared to 3‰ salinity. (**D**) Enriched GO terms from significantly differential APA genes in 60‰ compared to 30‰ salinity.

The analysis of alternative polyadenylation (APA) revealed distinct patterns in different salinity conditions. For 30‰ vs. 3‰ salinity, 17 transcripts preferred distal PAS positions, and 38 transcripts favored proximal PAS positions. For the 60‰ vs. 30‰ comparison, 41 transcripts utilized distal polyadenylation site (PAS) positions, while 27 transcripts used proximal PAS positions. For 60‰ vs. 3‰ salinity, 23 transcripts opted for distal PAS positions, while 33 transcripts utilized proximal PAS positions (Figure 4A). Genes with significant APA events in the 30‰ vs. 3‰ group tended to favor short APA under 30‰ salinity conditions, in line with 60‰ vs. 3‰, whereas, in the 60‰ vs. 30‰ group, *L. marina* tended to preferentially select distal polyadenylation site (PAS) positions, leading to the formation of long 3′ UTR transcripts in response to high salinity stress (Figure 4B). Further analysis using the Pearson correlation coefficient (r) revealed that both low and high salt induced a bimodal response in the 3′ UTR’s length change (Figure 4B). Details of the APA events are presented in Supplementary Table S19.

Next, we performed a GO analysis for the genes containing APA (55, 56, and 68 in 30‰ vs. 3‰, 60‰ vs. 3‰, and 60‰ vs. 30‰ salinity, respectively) (Figure 4A). We observed that GO terms such as single-organism cellular process, signal transduction, intracellular membrane-bounded organelles, and DNA binding were enriched in 30‰ compared to 3‰ salinity (Figure 4C, Supplementary Table S20), while GO terms including transmembrane receptor activity, signal transduction, protein kinase activity, ion transmembrane transporter activity, ion channel activity, and DNA binding were enriched in 60‰ compared to 30‰ salinity (Figure 4D, Supplementary Table S21). Genes related to the GO term “DNA binding” (*ceh-32* and *pal-1*) showed different APA usages in both 60‰ vs. 30‰, and 30‰ vs. 3‰ salinity, indicating a regulating role of TFs in response to salinity stress. *EVM0010823/rpoa-1* had a lengthening 3′ UTR in 60‰ compared to 30‰ salinity. Strikingly, we found that *nhr-31*, which encodes an essential gene for resistance to osmotic stress in *C. elegans* [37], exhibited a lengthening 3′ UTR in 30‰ compared to 3‰ salinity.

## 4. Discussion

### 4.1. DEGs from ONT lrRNA-seq Are Highly Correlated with Illumina Data

Through Illumina RNA sequencing, Xie et al. recently reported *L. marina*’s transcriptional responses to different salinity conditions (3‰, 30‰, and 60‰) [1], in line with the three salinities deployed in this study. The DEGs obtained from the current ONT lrRNA-seq were highly correlated with those identified in our previously reported Illumina short-read RNA sequencing data (Supplementary Figures S1 and S16). Accordingly, we found many overlapped GO terms between this study and our previously reported Illumina data [1] (Supplementary Figures S2–S4). For instance, when comparing the 30‰ to the 3‰ salinity condition, GO terms such as oxidoreductase activity, cation transmembrane transport, and ion transmembrane transport were shared between the ONT lrRNA-seq and Illumina data (Supplementary Figure S2). Similarly, GO terms including extracellular space, the structural constituent of cuticle, substrate-specific channel activity, ion transport, and substrate-specific transmembrane transporter activity were shared between the ONT and Illumina short-read sequencing, when comparing 60‰ to 30‰ salinity (Supplementary Figure S3). In addition, for the 60‰ vs. 3‰ salinity comparison, GO terms such as structural the constituent of cuticle, ion transport, transmembrane transport, ATP hydrolysis-coupled proton transport, and the proton-transporting V-type ATPase complex were enriched for both the ONT lrRNA-seq in this study (Supplementary Figure S4) and our previously reported Illumina short read RNA sequencing [1].

### 4.2. Upregulated and Downregulated DEGs When Salinity Increased, Revealed by lrRNA–seq

We found 79 upregulated DEGs that were shared by all three salinity comparisons (Figure 1B), which means that these 79 genes were significantly increased when the salinity increased (Supplementary Figure S5). The GO analysis of the above 79 DEGs showed that terms such as DNA binding and the structural constituent of cuticle were significantly enriched (Supplementary Table S9). “DNA binding”-related genes (*daf-16*, *ceh-10*, *elt-6,* and *zfh-2*) and “structural constituent of cuticle”-related genes (*col-107*, *ttll-4*, *col-34,* and *dpy-13*) exhibited upregulation with increasing salinity (Supplementary Figure S5). It was reported that the targets of DAF-16 protected *C. elegans* from extreme hypertonic stress [38].

In addition, we observed 119 downregulated DEGs that were shared by all three salinity comparisons (Figure 1C), which means that these 119 genes were significantly decreased when the salinity increased (Supplementary Figure S6). Through the GO analysis of the above 119 DEGs, we found that GO terms including acid phosphatase activity, hydrolase activity–acting on ester bonds, and ion transport were enriched (Supplementary Table S10). “Acid phosphatase activity”-related genes (*pho-6*, *pho-8*, *pho-14,* and *Y105C5B.15*) and “ion transport”-related genes (*lgc-9*, *lgc-15*, *lgc-18*, *lgc-47*, *nkcc-1*, *acd-5*, *pitr-5,* and *aat-8*) exhibited downregulation with increasing salinity (Supplementary Figure S6). *EVM0005869/pho-8*, *EVM0011280/pho-14*, *EVM0008010/nkcc-1*, *EVM0012480/acd-5*, and *EVM0012566/pitr-5* were also identified in the downregulated DEGs of our previous Illumina data, when comparing the 30‰ to 3‰ salinity condition. Accordingly, *EVM0016464/lgc-9* and *EVM0017483/aat-8* were also identified in the downregulated DEGs of the Illumina data for the 60‰ vs. 30‰ salinity comparison.

### 4.3. DAS Identification under Different Salinity Conditions

Through the GO enrichment analysis of 214 genes containing DAS in 30‰ compared to 3‰ salinity, we found that GO terms such as cellular component assembly and coenzyme biosynthetic process were enriched (Supplementary Table S12). “Cellular component assembly”-related genes (*luc-7L*, *luc-7L3*, *hil-1*, *cav-1*, *B0035.15*, *F46G10.1*, and *VM106R.1*) and “coenzyme biosynthetic process”-related genes (*sams-4*, *Y65B4A.8,* and *tpk-1*) showed differential alternative splicing. In addition, we observed that GO terms such as cellular component assembly and coenzyme biosynthetic process were also enriched in 60‰ compared to 30‰ salinity (Supplementary Table S13). Furthermore, we observed that 86, 125, and 81 genes that contained DAS were DEGs, for comparisons between 30‰ and 3‰, 60‰ and 30‰, and 60‰ and 3‰, respectively (Figure 2B). Through the GO enrichment analysis of the 86 genes containing DAS in 30‰ compared to 3‰ salinity, we found that GO terms such as the nucleus, ion transport, and aromatic compound biosynthetic process were enriched (Supplementary Table S15). “nucleus”-related genes (*Y39E4B.6*, *mdt-26*, *luc-7L*, *luc-7L3*, and *hil-1*), “ion transport”-related genes (*atp-2*, *pitr-1*, *nhx-8*, *vha-12*, and *mod-1*), and “aromatic compound biosynthetic process”-related genes (*Y39E4B.6*, *lsy-12*, *tpk-1*, and *elt-3*) showed both differential alternative splicing and differential gene expression. Through the GO enrichment analysis of the 125 genes containing DAS in 60‰ compared to 30‰ salinity, we observed that GO terms including aromatic compound biosynthetic process, signal transduction, and ion transmembrane transporter were enriched (Supplementary Table S16). “Aromatic compound biosynthetic process”-related genes (*tpk-1*, *pnk-4*, *Y39E4B.6*, *gcy-31*, *nhr-3*, and *nhr-43*), “signal transduction”-related genes (*exc-5*, *gcy-31*, *nhr-3*, *nhr-43*, *ser-1*, and *rhgf-2*), and “ion transmembrane transporter”-related genes (*nipa-1*, *F23F1.6*, *del-5*, *abts-4*, and *aat-8*) exhibited both differential alternative splicing and differential expression. *Luc-7L* and *luc-7L3* belong to the LUC7-related splicing factor homolog gene family. Studies indicate that the human homolog genes LUC7L3–LUC7L form a complex, binding with U1 snRNP, mediating splicing at the 5′ splice site and influencing intron retention [39]. LUC7L3 plays a role in inhibiting hepatitis B virus replication [40] and regulating various development-related AS events [41]. In this study, the expression of *EVM0016785/luc-7L* and *EVM0002988/luc-7L3* was significantly downregulated, and those two genes underwent SE and RI events, respectively. Furthermore, a study has reported that *hil-1* is a direct target of *daf-16* [42], of which the expression level was observed to be significantly increased as the salinity increased in this study (Supplementary Figure S5). Thus, we inferred that *hil-1* might play an essential role in the response to high-salinity conditions. Additionally, *atp-2*, by encoding a subunit of ATPase, modulates the function of the mitochondrial oxidative respiratory chain [43], which might be involved in energy regulation in response to salinity stresses. NHR-23 regulates molting and lipid transport/metabolism in *C. elegans* and contributes to apical extracellular matrix (aECM) remodeling [44]. Our data suggested that *nhr-23* and *nhr-32* specifically responded to the 60‰ salinity condition. In addition, *tpk-1*, by encoding the ortholog of human TPK1 (thiamin pyrophosphokinase 1) gene, modulates the activity of all thiamine-dependent enzymes [45]. Our data suggested that both the splicing and expression of *tpk-1* might play essential roles in the response to salinity stresses.

### 4.4. The Marine Nematode Exhibits Distinct APA Usage under Different Salinity Conditions

Previous studies have revealed that the regulatory patterns of APA events vary under different conditions [46,47,48,49,50]. It was reported that there was a gradual increase in 3′ UTR length during embryonic development [51]. Conversely, in the proliferation of CD4^+^ T cells, a preference for using short 3′ UTRs was observed [52], suggesting a preference for long APA during development and short APA during cell proliferation [53]. Cancer cells also exhibited an increased usage of short 3′ UTRs [54,55,56,57,58]. The occurrence of both significant long and short APA events in our APA results indicates that salinity stress has multiple and complex effects on transcriptional regulation in organisms.

Although the APA dynamics in response to stresses have been well studied in plants, few APA analyses have been conducted on marine invertebrates, especially the marine nematode [34]. Here, to the best of our knowledge, we are the first to report alternative polyadenylation usage in marine nematodes under different salinity conditions. Of all the enriched GO terms (Figure 4C, Supplementary Table S20), we observed that “single-organism cellular process”-related genes (*famp-1*, *T23B5.4,* and *EVM0007151*), “signal transduction”-related genes (*plx-1*, *D1014.2,* and *nhr-31*), “intracellular membrane-bounded organelle”-related genes (*nhr-31*, *EVM0002118,* and *EVM0012000*), and “DNA binding”-related genes (*nhr-31*, *ceh-32,* and *pal-1*) exhibited differential alternative polyadenylation in 30‰ compared to 3‰ salinity. Of those ten genes, three genes (*D1014.2*, *T23B5.4*, *EVM0012000*) had elongated 3′ UTRs and seven (*nhr-31*, *ceh-32*, *pal-1*, *plx-1*, *famp-1*, *EVM0007151*, *EVM0002118*) had shortened 3′ UTRs (Supplementary Table S20). In addition, of the enriched GO terms (Figure 4D, Supplementary Table S21), we found that “transmembrane receptor activity”-related genes (*acr-10*, *npr-21*, *pdfr-1*, *frpr-5,* and *EVM0006162*), “signal transduction”-related genes (*npr-21*, *pdfr-1*, *frpr-5,* and *pkn-1*), “protein kinase activity”-related genes (*unc-51*, *ZK596.2*, *M03C11.1,* and *pkn-1*), “ion transmembrane transporter activity”-related genes (*asic-2*, *acr-10*, *zipt-16*, *nca-2,* and *EVM0006162*), “ion channel activity”-related genes (*asic-2*, *acr-10*, *nca-2,* and *EVM0006162*), and “DNA binding”-related genes (*rpoa-1*, *ceh-32*, *vab-15*, *pal-1,* and *EVM0015267*) exhibited differential alternative polyadenylation in the 60‰ compared to the 30‰ salinity environment (Figure 4D, Supplementary Table S21). Thirteen (*asic-2*, *unc-51*, *acr-10*, *npr-21*, *M03C11.1*, *rpoa-1*, *ceh-32*, *vab-15*, *pal-1*, *frpr-5*, *nca-2*, *EVM0006162*, *EVM0015267*) of those above seventeen genes showed elongated 3′ UTRs, while four genes (*pdfr-1*, *ZK596.2*, *zipt-16,* and *pkn-1*) exhibited shortened 3′ UTRs in 60‰ compared to 30‰ salinity. To be specific, all three genes (*nhr-31*, *ceh-32,* and *pal-1*) enriched in “DNA binding” in 30‰ vs. 3‰ salinity had shortened 3′ UTRs, while all five genes (*rpoa-1*, *ceh-32*, *vab-15*, *pal-1,* and *EVM0015267*) enriched in “DNA binding” in 60‰ vs. 30‰ salinity had elongated 3′ UTRs. In addition, four (*sic-2*, *acr-10*, *nca-2,* and *EVM0006162*) out of five genes enriched in “ion transmembrane transporter activity” in 60‰ vs. 30‰ also had elongated 3′ UTRs despite the other one gene, *zipt-16,* having a shortened 3′ UTR. As expected, *zipt-16* was also a significantly upregulated gene in 60‰ vs. 30‰ salinity (Supplementary Table S1), and has been reported to have interactions with *daf-16* and *skn-1* [59]. The two transcripts of *zipt-16* (*EVM0008784.1* and *EVM0008784.2*) were also significantly upregulated in 60‰ vs. 30‰ salinity (Supplementary Table S22). Furthermore, *asic-2*, *acr-10,* and *nca-2* were all significantly downregulated in 60‰ vs. 30‰ salinity, indicating that their expression might be regulated by APA. Additionally, we found that homeodomain proteins including WNT-signaling-associated *EVM0013683/pal-1*, *EVM0012598/ceh-32,* and *EVM0013568/vab-15* might contribute to salinity stress resistance [60]. We also provided new insight into how *nhr-31* improves the nematode’s ability to survive acute osmotic stress. The shortened *EVM0011368/nhr-31* was also a significantly upregulated DEG in 30‰ vs. 3‰ salinity (Supplementary Table S1), indicating its role in the acute osmotic stress response. Collectively, our data provided a more comprehensive perspective for investigating the mRNA dynamics in response to salinity stress.

## 5. Conclusions

In this study, for the first time, to our knowledge, we deployed ONT long-read sequencing to characterize the transcriptome responses to different salinity conditions of marine nematodes. We found that the DEGs obtained from the current lrRNA-seq were highly correlated with Illumina data from our previous report. In addition, we found the expression of 79 genes was significantly increased, while 119 genes were significantly repressed, as salinity increased. Furthermore, we found that 86, 125, and 81 genes that contained DAS were also DEGs, for comparisons between 30‰ and 3‰, 60‰ and 30‰, and 60‰ and 3‰, respectively. Moreover, we demonstrated the landscape of alternative polyadenylation in marine nematodes under different salinity conditions. This study provides novel insights for further investigation into the mechanisms underpinning euryhalinity and might also contribute to the study of salinity stresses induced by global climate change.

## Figures and Tables

**Table 1:** Summary of ONT lrRNA sequencing in different salinity conditions.

	Million Reads	Mean Length	Number of Isoform/Gene	Number of Novel Isoform/Gene
3‰	5.34	737.80	13,769/10,985	3161/2549
30‰	5.01	903.60	14,098/10,954	3538/2800
60‰	6.25	753.91	13,920/11,083	3226/2594

## Data Availability

The raw ONT lrRNA sequencing data in this study were deposited in NCBI (PRJNA1072286).

## Supplementary Materials

The following supporting information can be downloaded at: https://www.mdpi.com/article/10.3390/genes15030317/s1.
