# Supplementary material for: Transcriptome Responses to Different Salinity Conditions in Litoditis marina, Revealed by Long-Read Sequencing
Source: Genes (Basel). 2024 Feb 28;15(3):317. doi: 10.3390/genes15030317 (PMC10970011; doi:10.3390/genes15030317)
Supplement: Supplementary file 1 [file genes-15-00317-s001.zip › Supplementary Information.pdf]

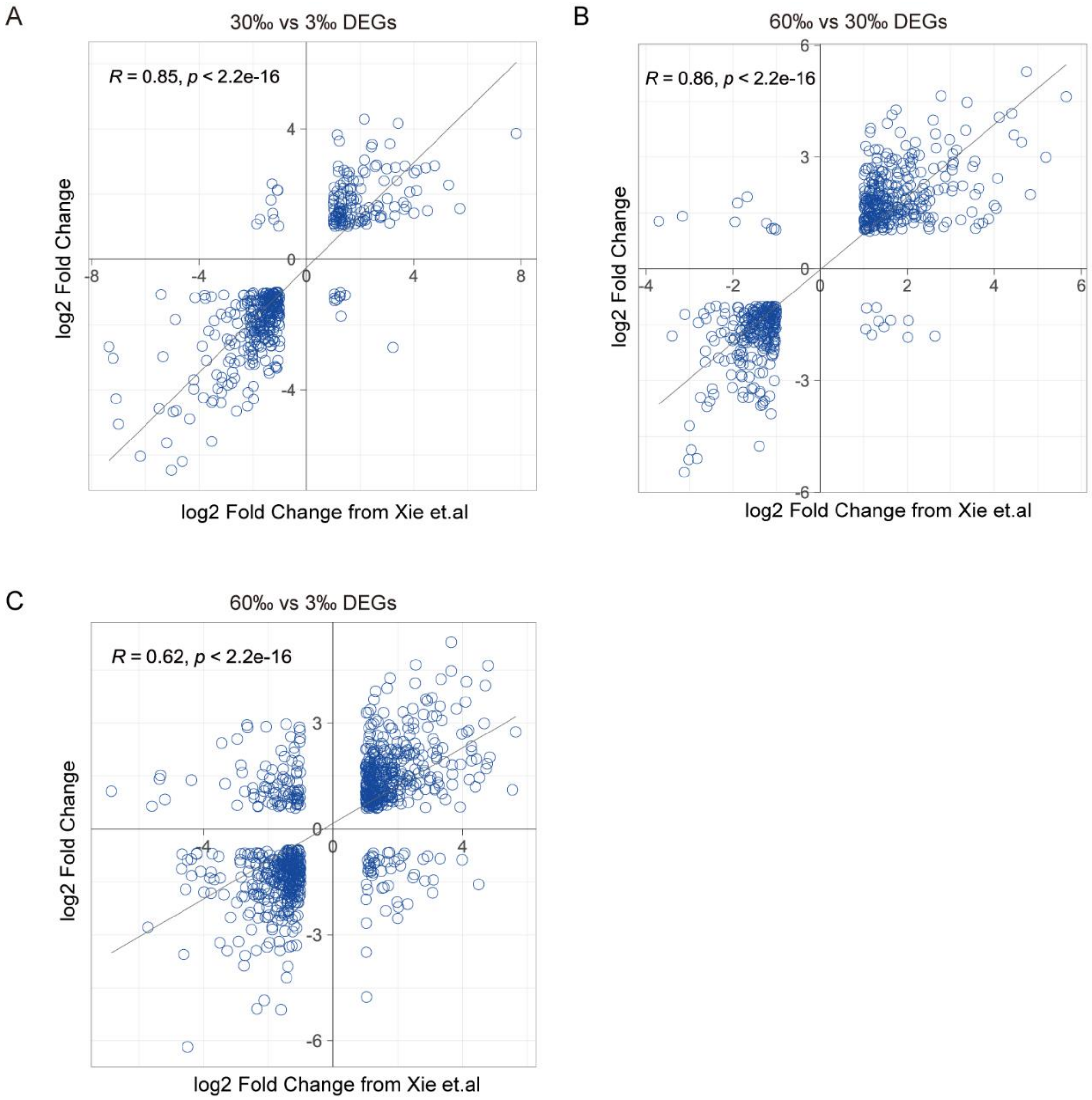

Figure S1. Comparison of DEGs between ONT and Illumina transcriptomes. A. Linear regression plot for the comparison of DEGs between 30‰ salinity and 3‰ salinity group. B. Linear regression plot for the comparison of DEGs between 60‰ salinity and 30‰ salinity group. C. Linear regression plot for the comparison of DEGs between 60‰ salinity and 3‰ salinity group. Comparison based on log2Fold Change of DEGs. Genes with FDR < 0.05 and fold change > 1.5 were considered as significantly differentially expressed.

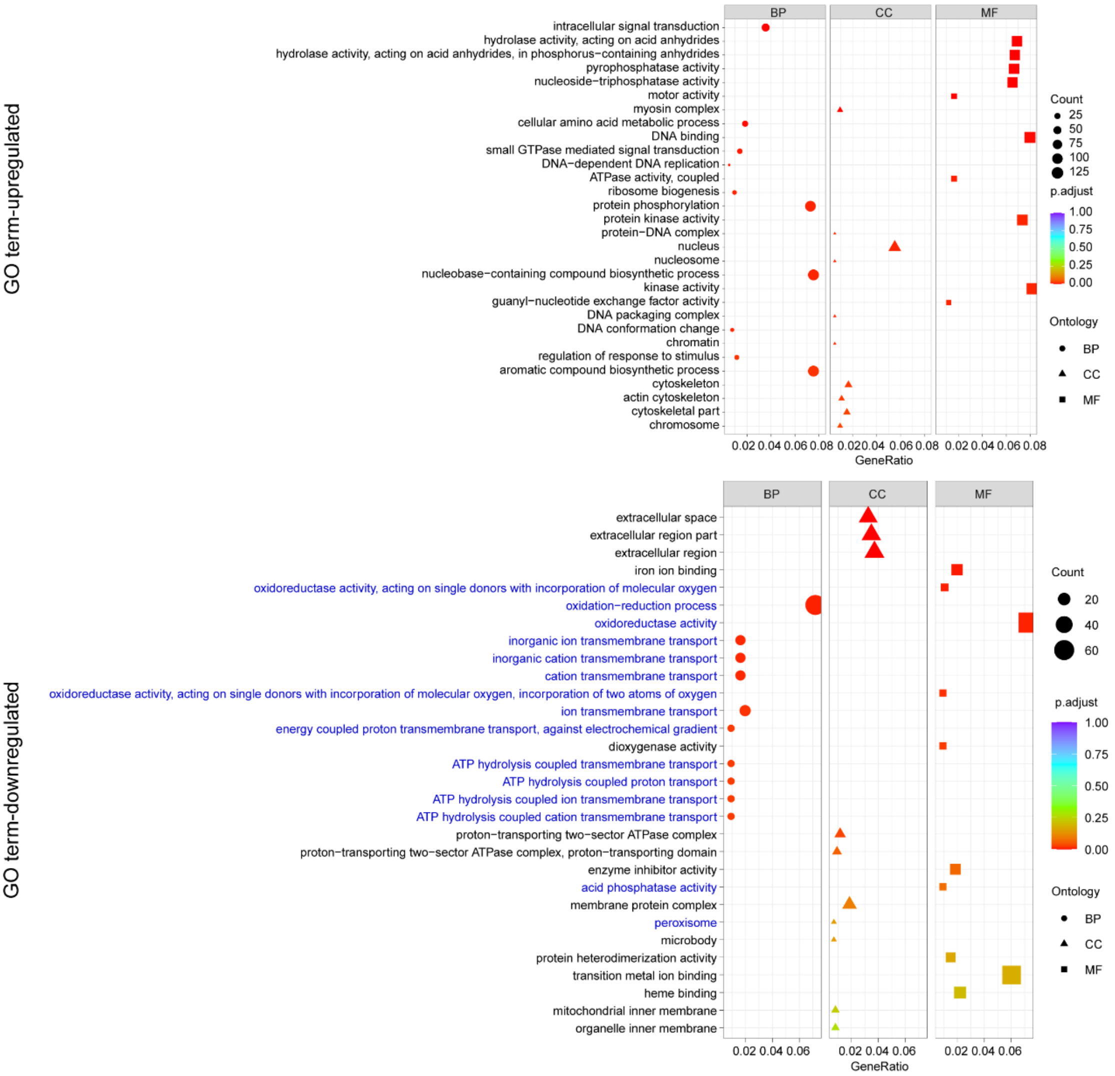

Figure S2. GO enrichment results of DEGs in the 30‰ vs 3‰ group. A. GO enrichment results of upregulated DEGs. B. GO enrichment results of downregulated DEGs. Blue terms indicate pathways similar to those enriched in Illumina DEGs.

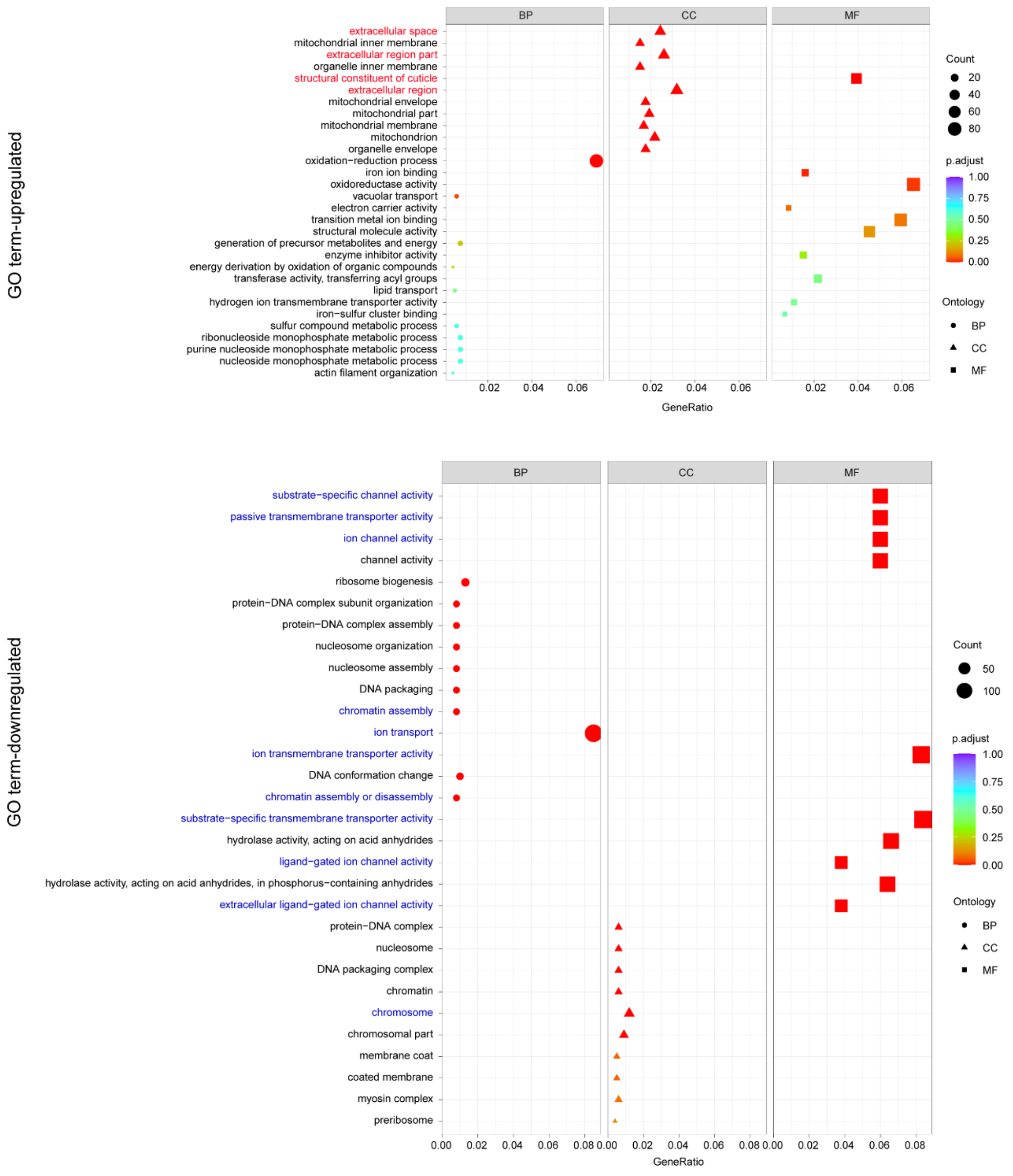

Figure S3. GO enrichment results of DEGs in the 60‰ vs 30‰ group. A. GO enrichment results of upregulated DEGs. Red terms indicate pathways similar to those enriched in Illumina DEGs. B. GO enrichment results of downregulated DEGs. Blue terms indicate pathways similar to those enriched in Illumina DEGs.

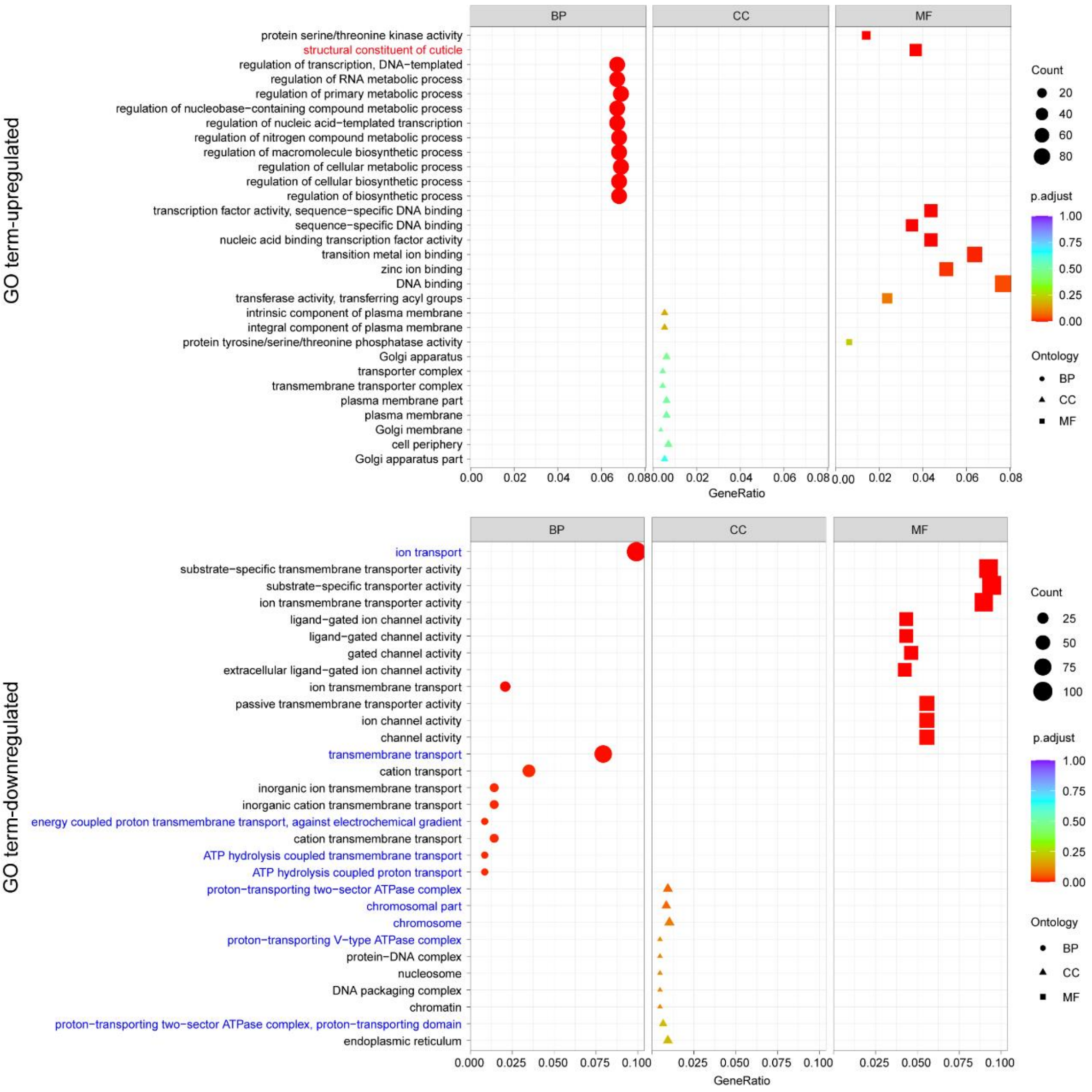

Figure S4. GO enrichment results of DEGs in the 60‰ vs 3‰ group. A. GO enrichment results of upregulated DEGs. Red terms indicate pathways similar to those enriched in Illumina DEGs. B. GO enrichment results of downregulated DEGs. Blue terms indicate pathways similar to those enriched in Illumina DEGs.

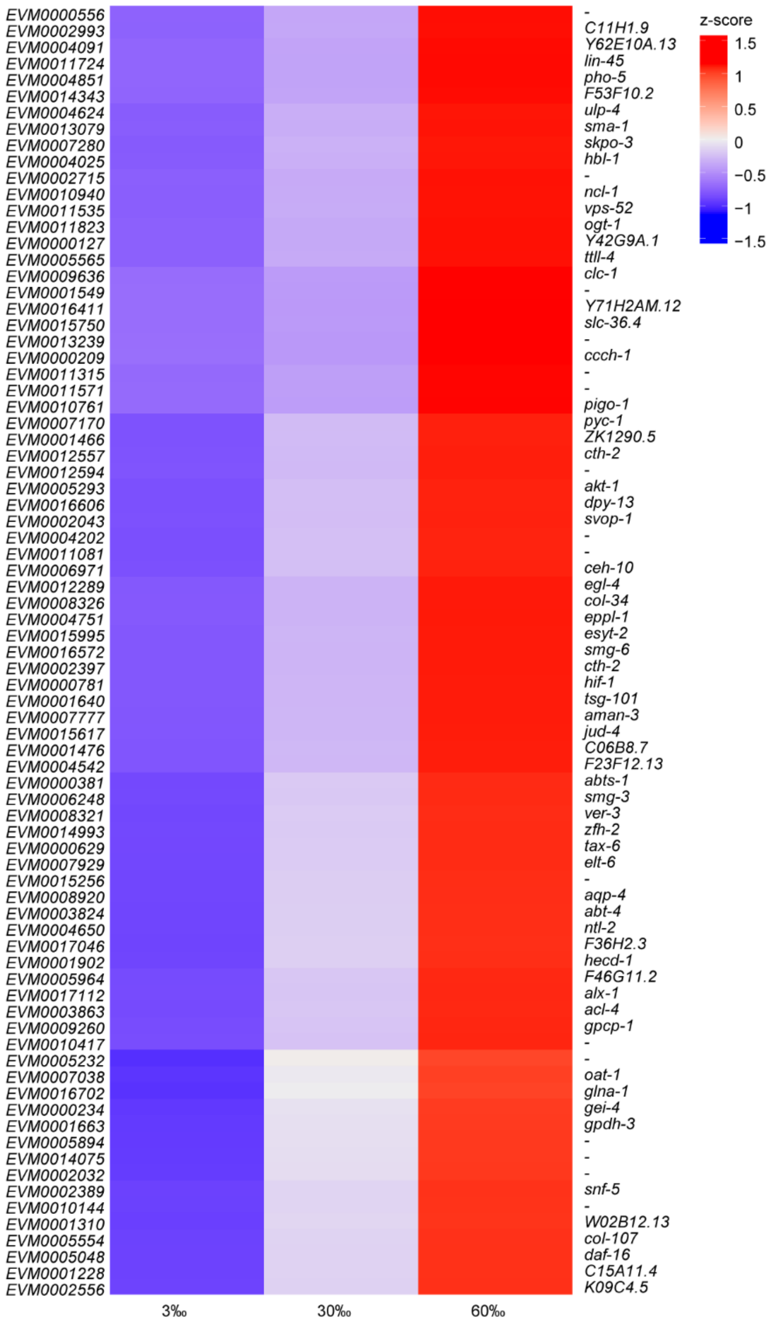

Figure S5. Heatmap depicting the expression levels of the overlapping 79 genes in the figure 1B for each comparison group.

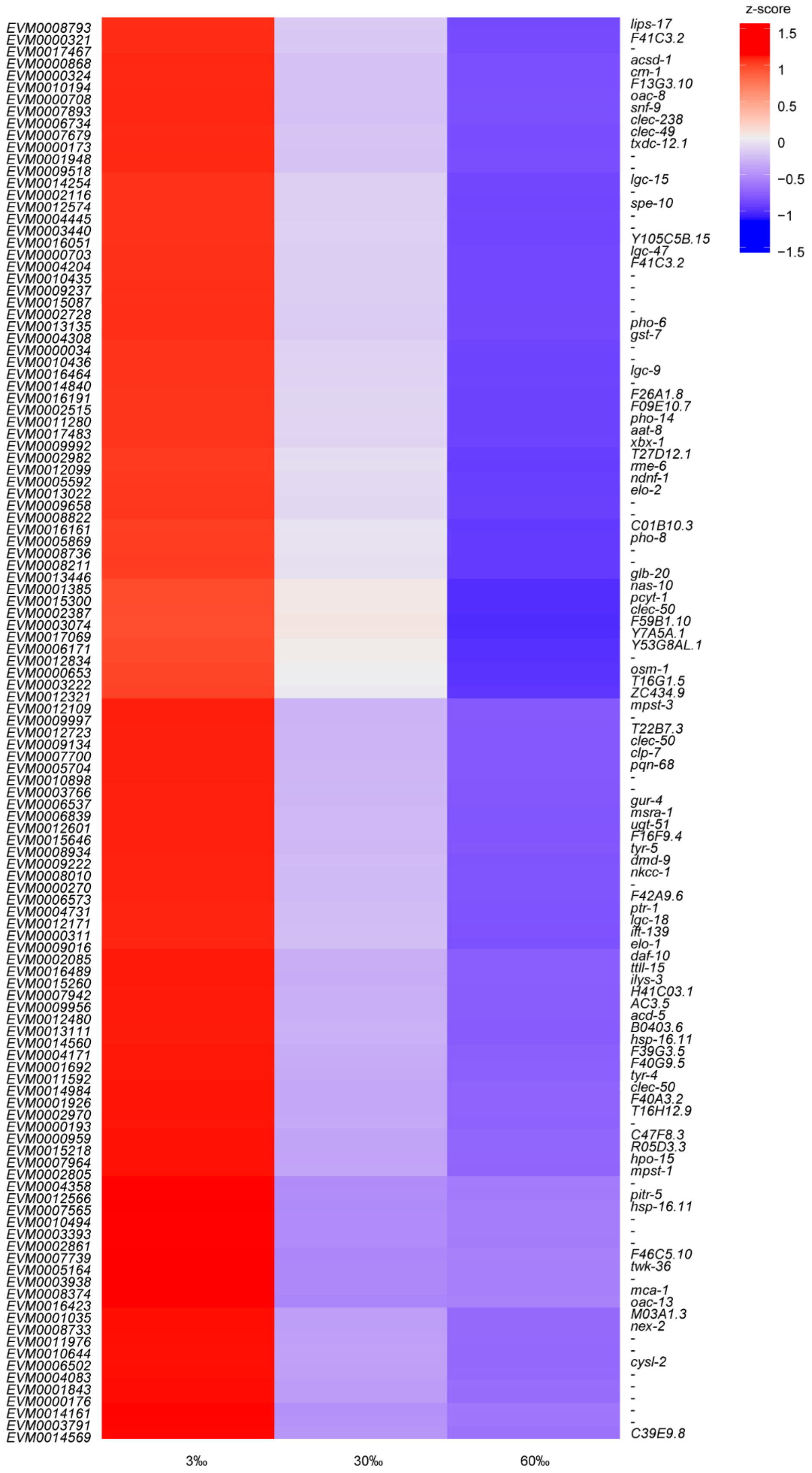

Figure S6. Heatmap depicting the expression levels of the overlapping 119 genes in the figure 1C for each comparison group.

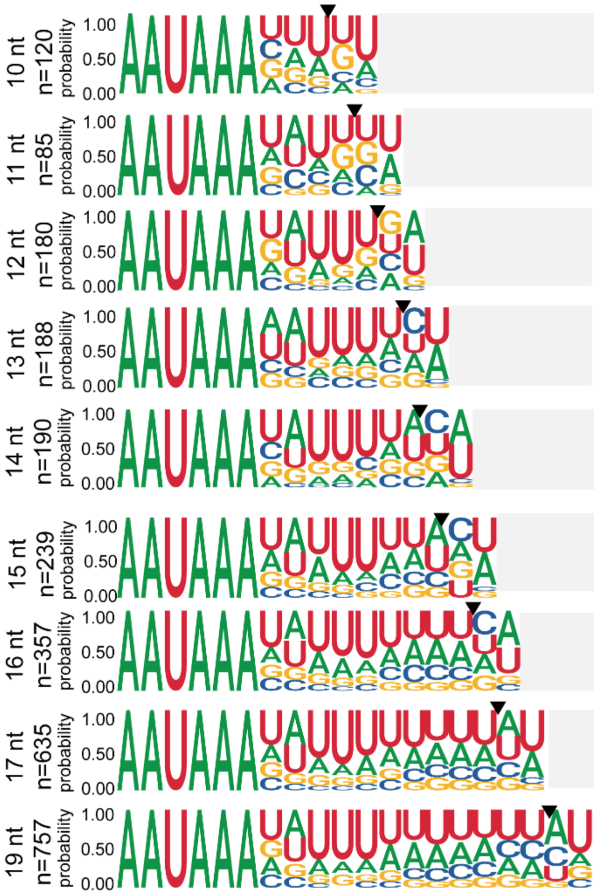

Figure S7. Sequence logos representing 10 to 19 nt (median distance from cleavage site to PAS motif) downstream of PAS motif in 3' UTRs under 3‰ salinity, except 18nt. Only the canonical motif AAUAAA is shown.

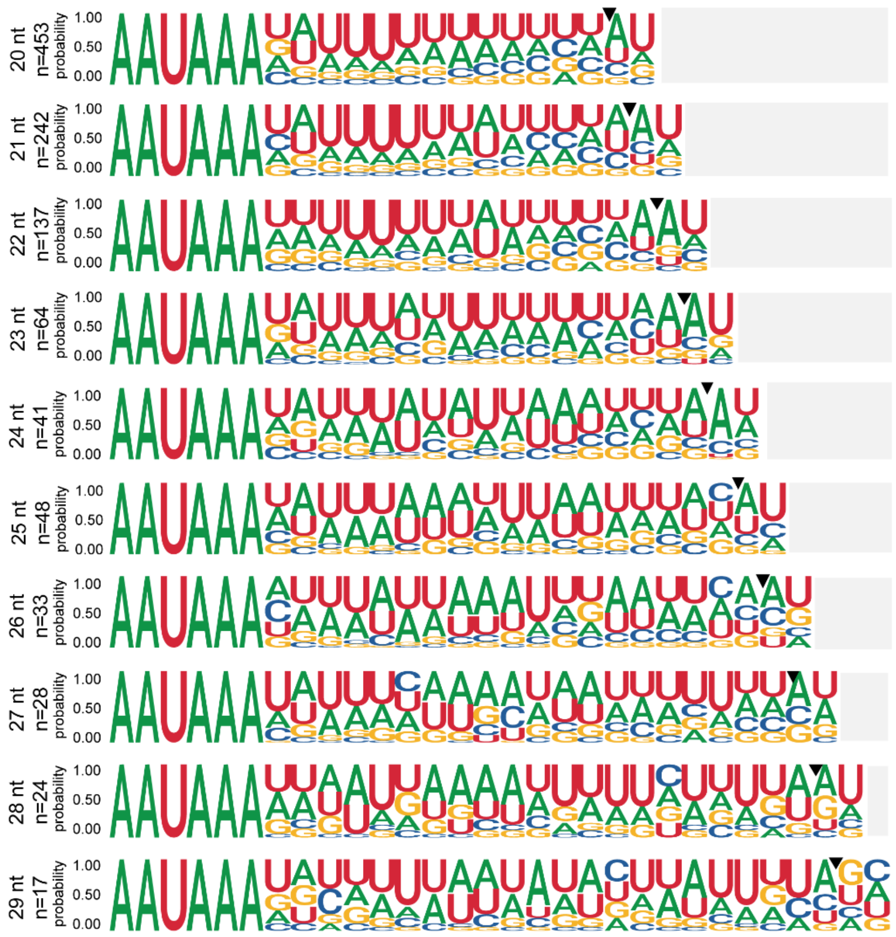

Figure S8. Sequence logos representing 20 to 29 nt (median distance from cleavage site to PAS motif) downstream of PAS motif in 3' UTRs under 3‰ salinity. Only the canonical motif AAUAAA is shown.

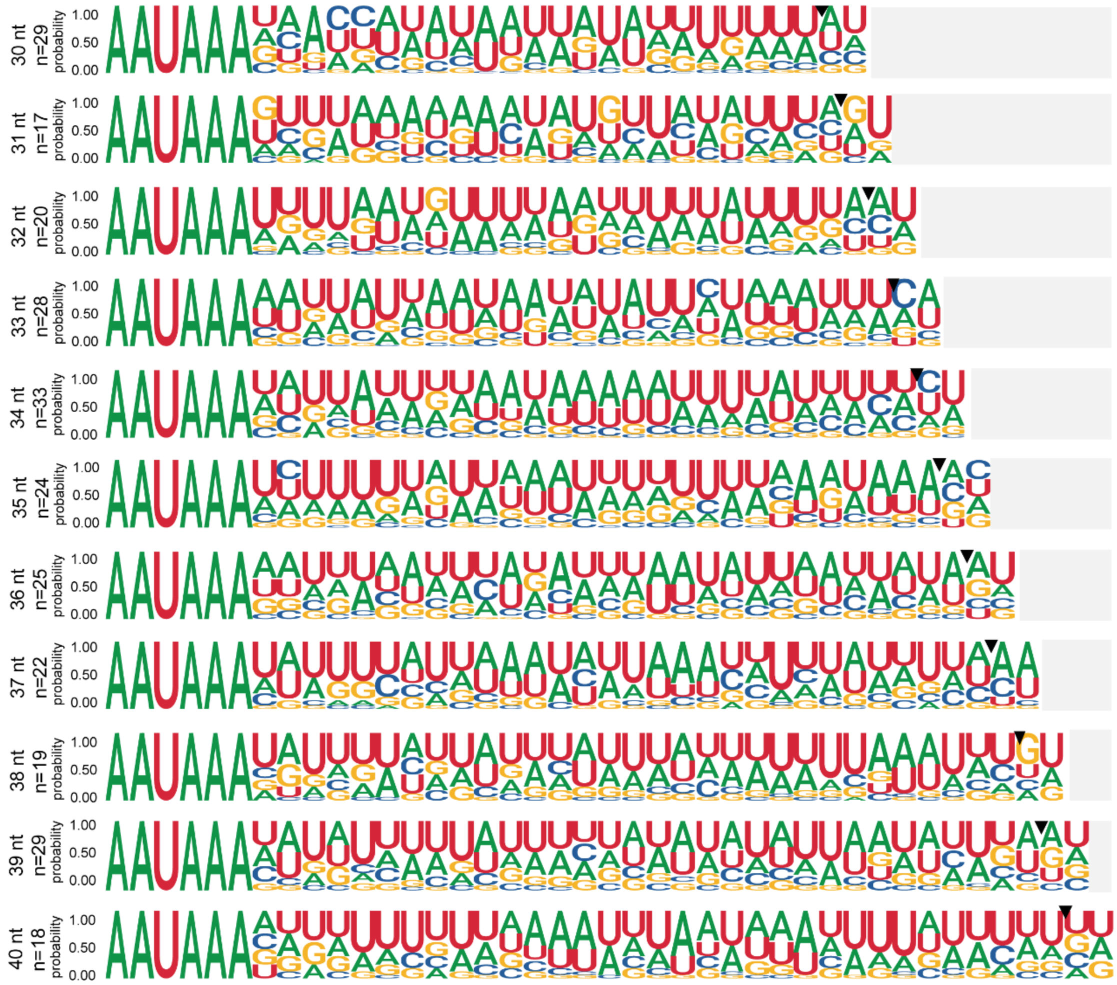

Figure S9. Sequence logos representing 30 to 40 nt (median distance from cleavage site to PAS motif) downstream of PAS motif in 3' UTRs under 3‰ salinity. Only the canonical motif AAUAAA is shown.

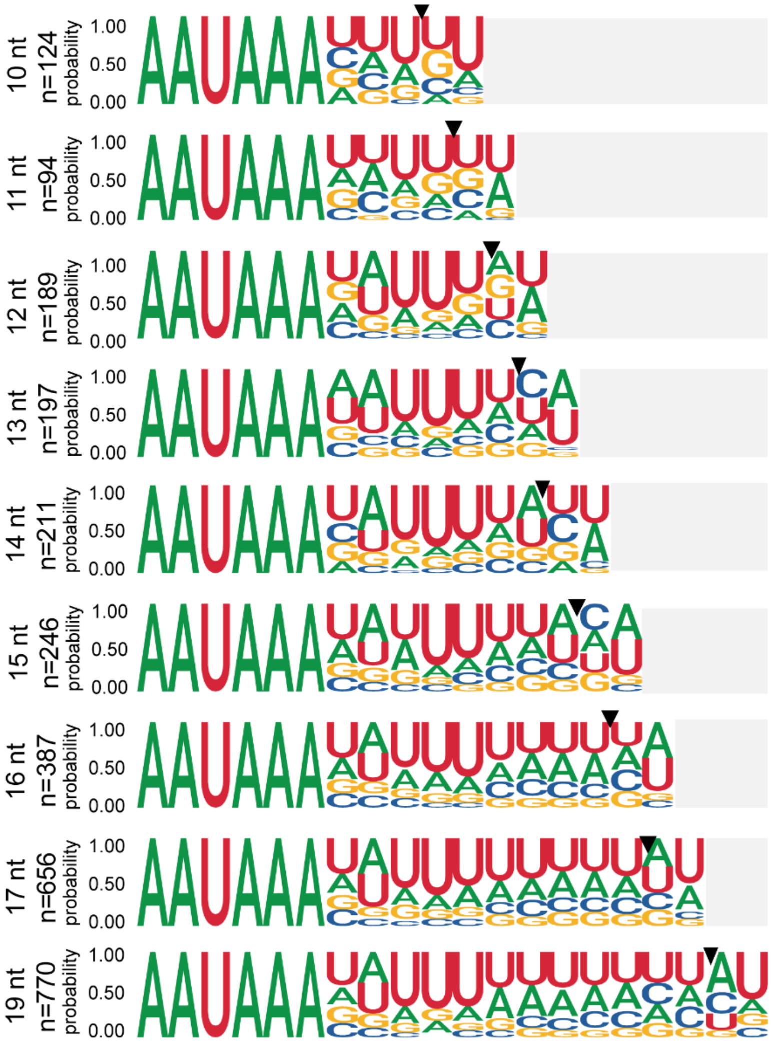

Figure S10. Sequence logos representing 10 to 19 nt (median distance from cleavage site to PAS motif) downstream of PAS motif in 3' UTRs under 30‰ salinity, except 18 nt. Only the canonical motif AAUAAA is shown.

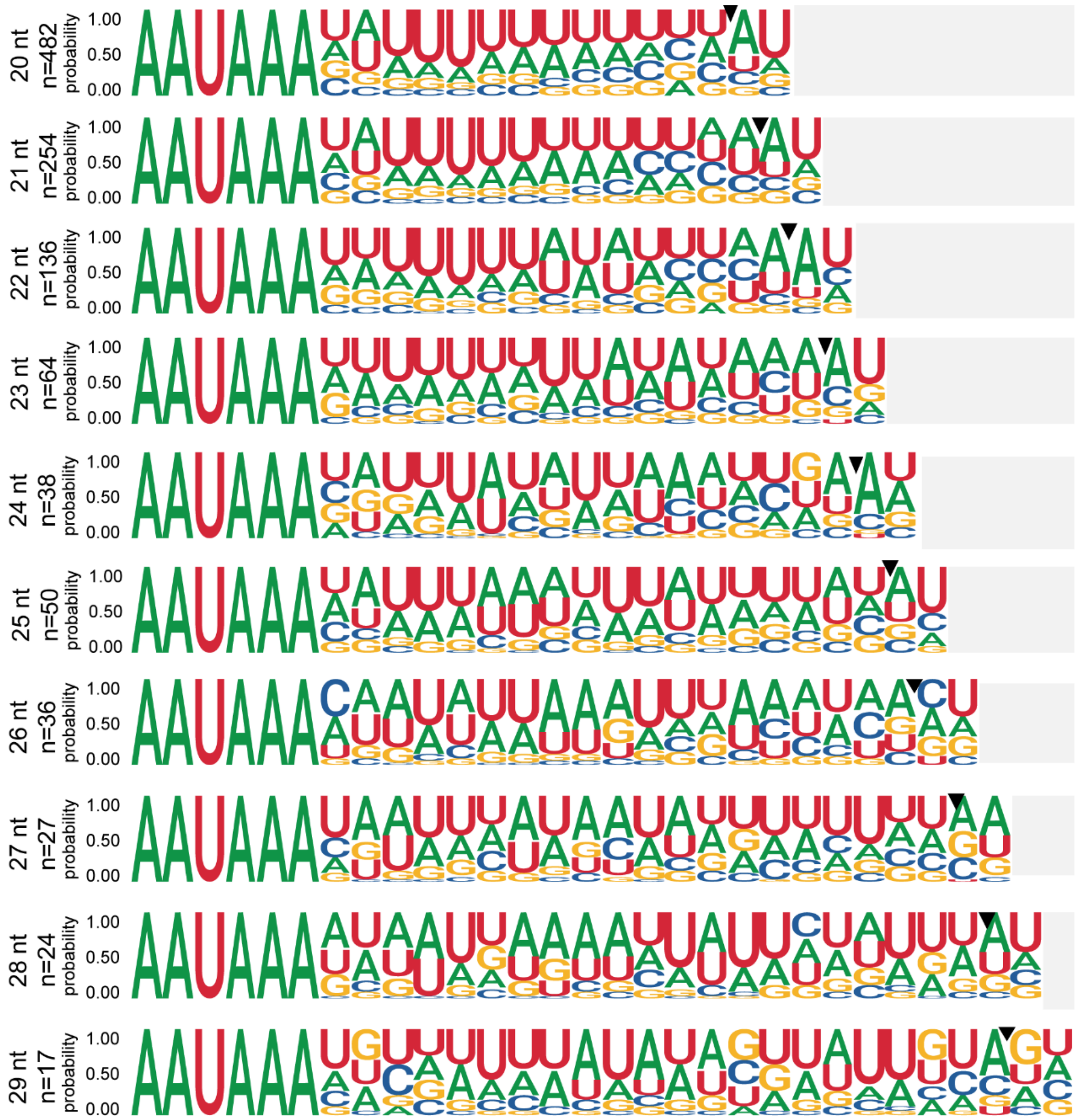

Figure S11. Sequence logos representing 20 to 29 nt (median distance from cleavage site to PAS motif) downstream of PAS motif in 3' UTRs under 30‰ salinity. Only the canonical motif AAUAAA is shown.

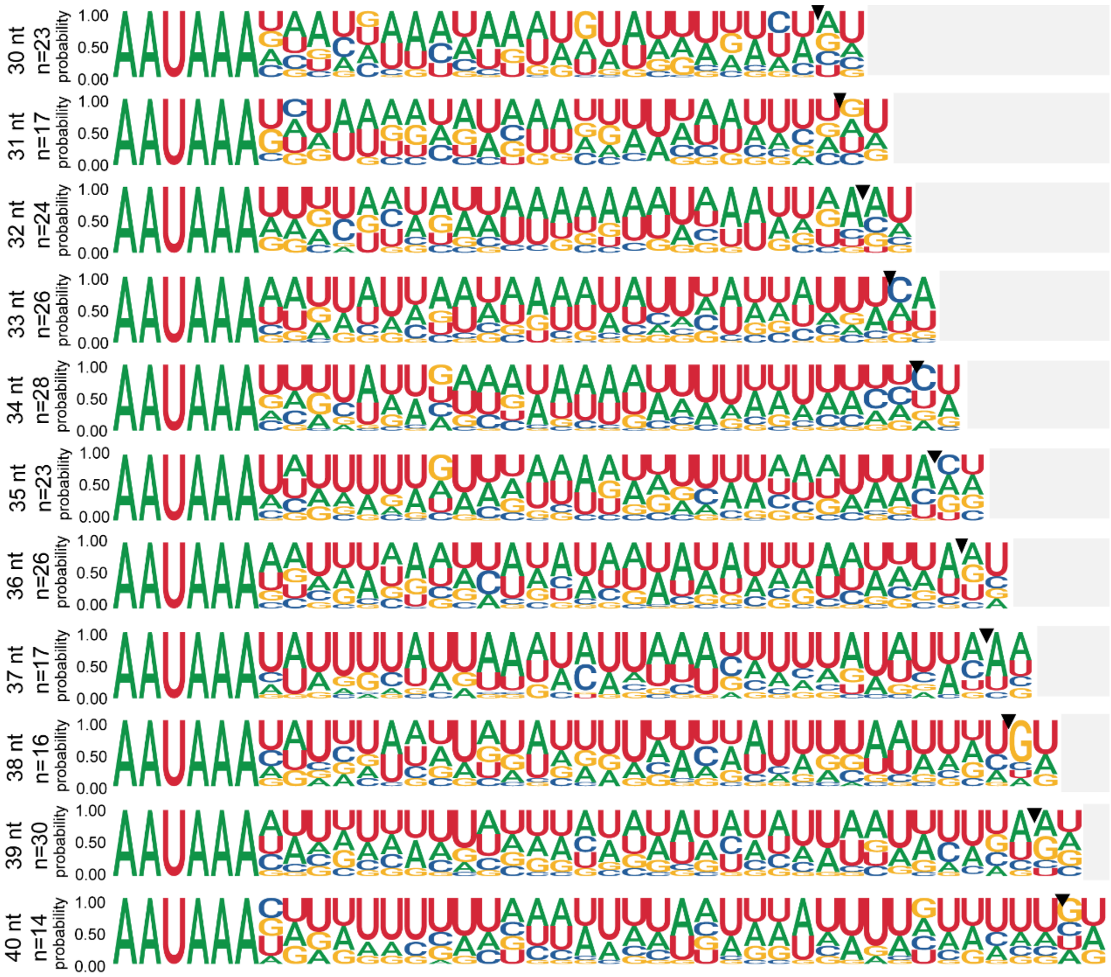

Figure S12. Sequence logos representing 30 to 40 nt (median distance from cleavage site to PAS motif) downstream of PAS motif in 3' UTRs under 30‰ salinity. Only the canonical motif AAUAAA is shown.

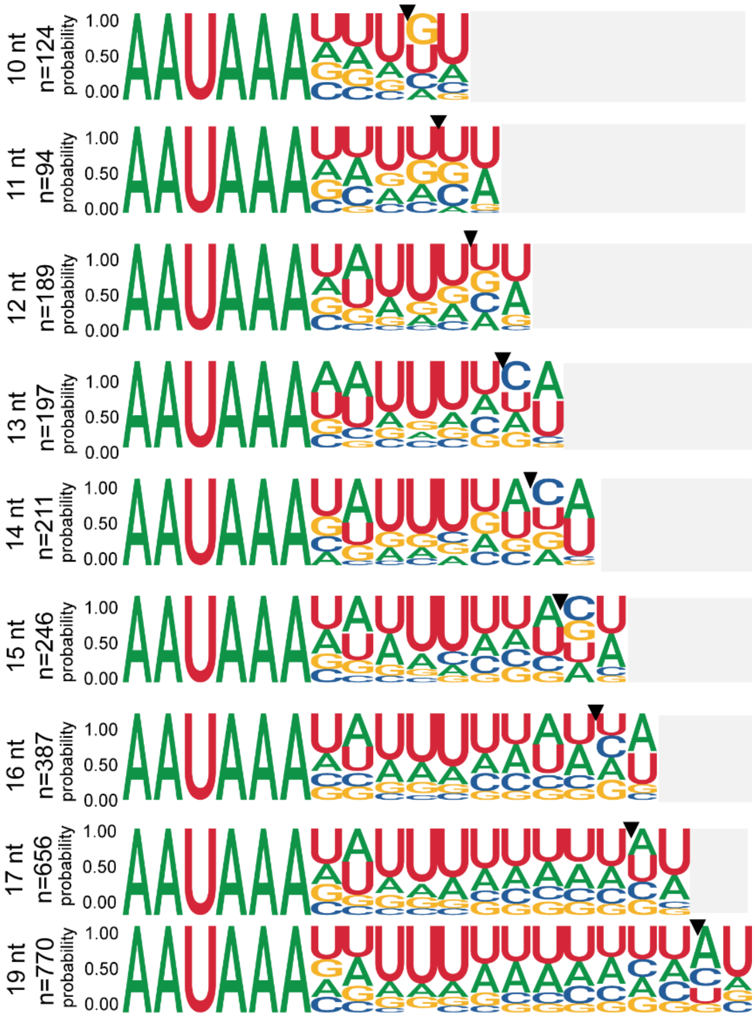

Figure S13. Sequence logos representing 10 to 19 nt (median distance from cleavage site to PAS motif) downstream of PAS motif in 3' UTRs under 60‰ salinity, except 18 nt. Only the canonical motif AAUAAA is shown.

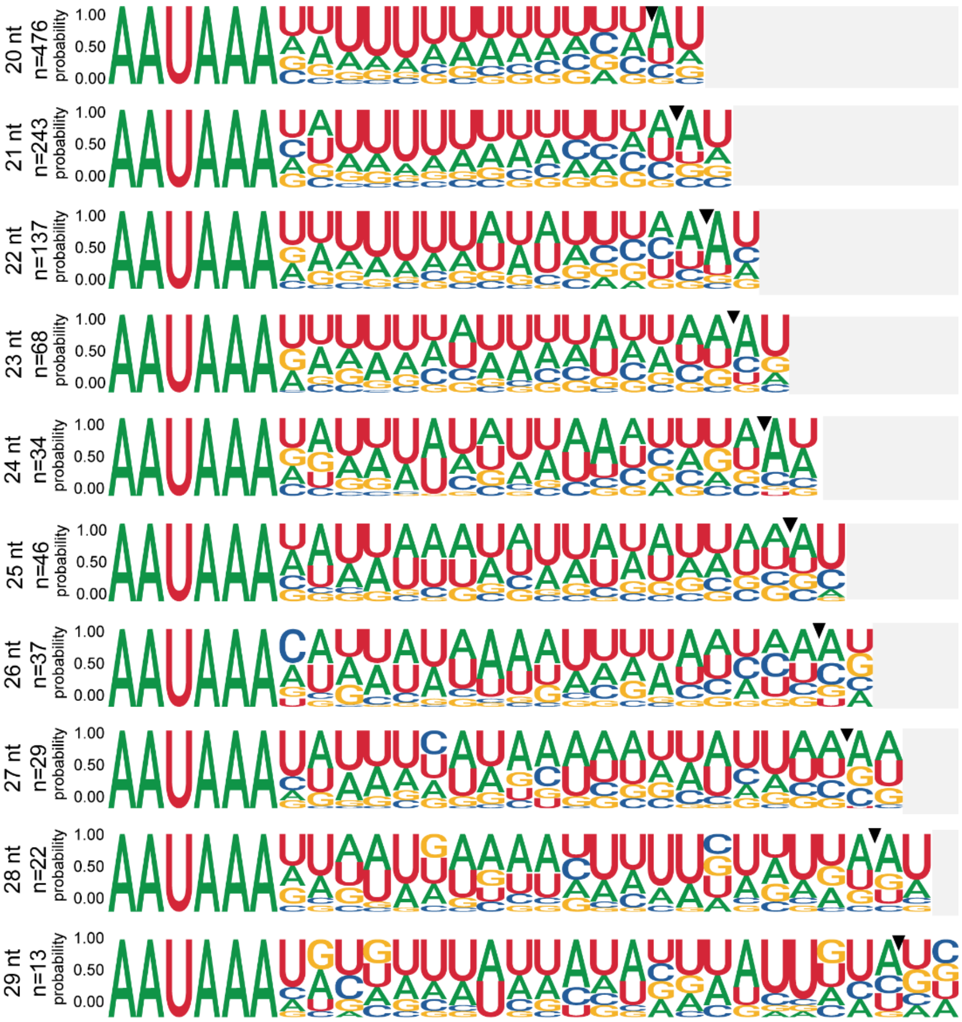

Figure S14. Sequence logos representing 20 to 29 nt (median distance from cleavage site to PAS motif) downstream of PAS motif in 3' UTRs under 60‰ salinity. Only the canonical motif AAUAAA is shown.

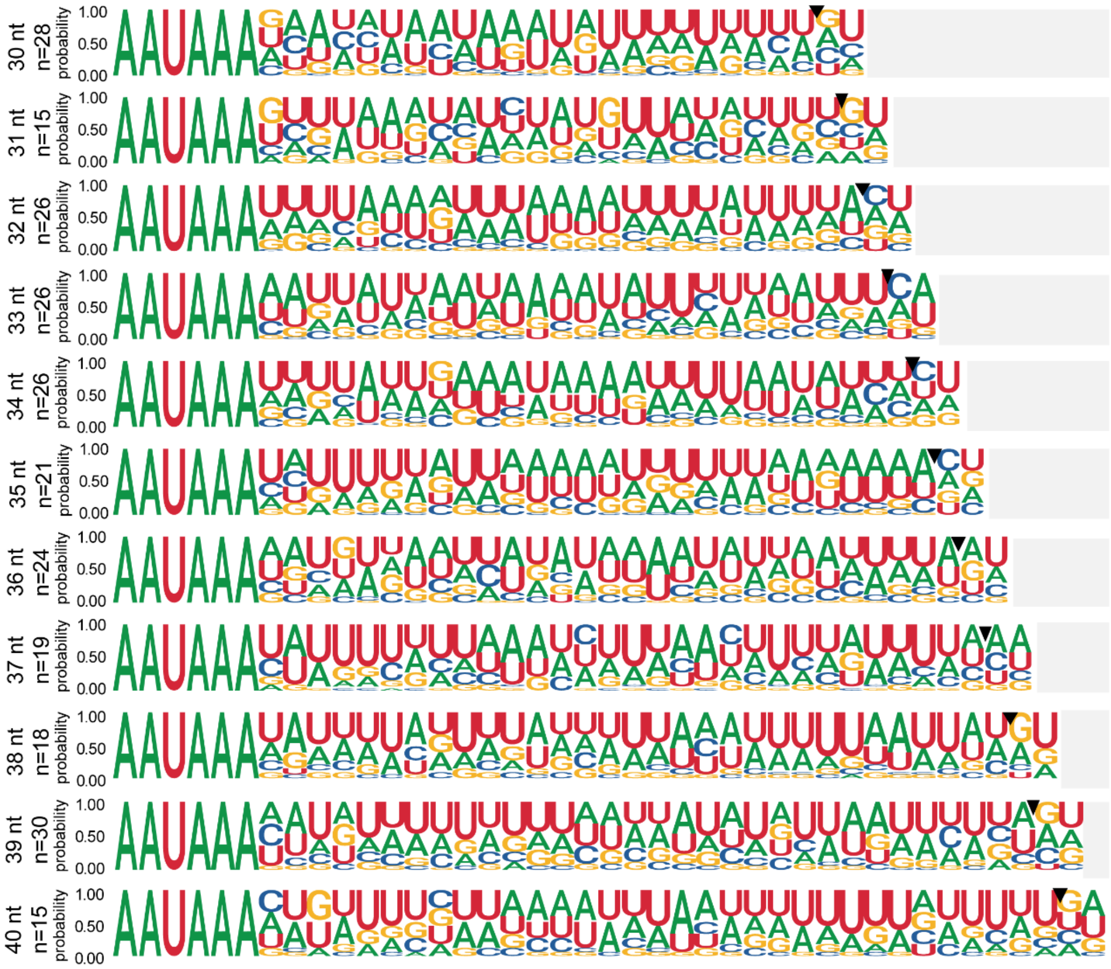

Figure S15. Sequence logos representing 30 to 40 nt (median distance from cleavage site to PAS motif) downstream of PAS motif in 3' UTRs under 60‰ salinity. Only the canonical motif AAUAAA is shown.

	30‰ vs. 3‰	60‰ vs. 3‰	60‰ vs. 30‰
Overlapped genes	1207	1906	1610
Xie's et al.	2473	3553	2779
this paper	5144	4721	6006

Figure S16. In comparing the DEGs from our study with those from the previous study using Illumina data.
